# Optical biopsy for esophageal squamous cell neoplasia by using endocytoscopy

**DOI:** 10.1186/s12876-022-02335-5

**Published:** 2022-05-21

**Authors:** Yoshihiko Shimoda, Yuichi Shimizu, Hiroaki Takahashi, Satoshi Okahara, Takakazu Miyake, Shin Ichihara, Ikko Tanaka, Masaki Inoue, Sayoko Kinowaki, Masayoshi Ono, Keiko Yamamoto, Shoko Ono, Naoya Sakamoto

**Affiliations:** 1 Department of Gastroenterology and Hepatology, Graduate School of Medicine and Faculty of Medicine, Hokkaido University, Sapporo, Hokkaido 060-8648 Japan; 2 Division of Endoscopy, Hokkaido University Hospital, Sapporo, Hokkaido 060-8648 Japan; 3 Department of Gastroenterology, National Hospital Organization, Hokkaido Medical Center, 5-7, Yamanote, Nishi-ku, Sapporo, 063-0005 Japan; 4 Department of Gastroenterology, Keiyukai Daini Hospital, Sapporo, Hokkaido 003-0027 Japan; 5 Department of Surgical Pathology, Sapporo Kosei General Hospital, Sapporo, Hokkaido 060-0033 Japan; 6 Department of Gastroenterology, Hokkaido University Hospital, Sapporo, Hokkaido 060-8648 Japan

**Keywords:** Diagnostic accuracy, Endocytoscopy, Esophageal cancer, Intraepithelial neoplasia, Optical biopsy

## Abstract

**Background:**

Endocytoscopy (ECS) enables microscopic observation in vivo for the gastrointestinal mucosa; however, there has been no prospective study in which the diagnostic accuracy of ECS for lesions that have not yet undergone histological diagnosis was evaluated. We conducted a surveillance study for patients in a high-risk group of esophageal squamous cell carcinoma (ESCC) and evaluated the in vivo histological diagnostic accuracy of ECS.

**Methods:**

This study was a multicenter prospective study. We enrolled 197 patients in the study between September 1, 2019 and November 30, 2020. The patients first underwent white light imaging and narrow band imaging, and ultra-high magnifying observation was performed if there was a lesion suspected to be an esophageal tumor. Endoscopic submucosal dissection (ESD) was later performed for lesions that were diagnosed to be ESCC by ECS without biopsy. We evaluated the diagnostic accuracy of ECS for esophageal tumorous lesions.

**Results:**

ESD was performed for 37 patients (41 lesions) who were diagnosed as having ESCC by ECS, and all of them were histopathologically diagnosed as having ESCC. The sensitivity [95% confidence interval (CI)] was 97.6% (87.7–99.7%), specificity (95% CI) was 100% (92.7–100%), diagnostic accuracy (95% CI) was 98.9% (94.0–99.8%), positive predictive value (PPV) (95% CI) was 100% (91.4–100%) and negative predictive value (NPV) (95% CI) was 98.0% (89.5–99.7%).

**Conclusions:**

ECS has a high diagnostic accuracy and there were no false positives in cases diagnosed and resected as ESCC. Optical biopsy by using ECS for esophageal lesions that are suspected to be tumorous is considered to be sufficient in clinical practice.

**Supplementary Information:**

The online version contains supplementary material available at 10.1186/s12876-022-02335-5.

## Background

Endoscopic diagnosis of early-stage gastrointestinal cancers has usually been performed by using conventional white-light endoscopy, chromoendoscopy, and image-enhanced magnification endoscopy. The accuracy of endoscopic diagnosis has been significantly improved. Esophageal squamous cell carcinoma (ESCC) is being detected more frequently in early stages. It has been reported that the accuracy of these endoscopic procedures for diagnoses of early-stage esophageal cancer is about 90% [1–3]. However, in actual clinical practice, endoscopic resection is usually performed after diagnosis of early esophageal cancer for histology of specimens obtained by endoscopic biopsy.

Recently, endocytoscopy (ECS), in which an endoscope with high resolution and ultra-high magnification is used, has been introduced, and it enables microscopic observation in vivo at the cellular level of the gastrointestinal mucosa [4, 5]. Inoue et al. developed an ECA classification for ESCC and dysplasia based on differences between the findings of ECS and histopathology [4]. They reported that ECA 4 and 5 correspond to histopathological grades 4 and 5 (equivalent to a lesion showing severe atypia that is indicated for treatment) in the Vienna classification [6, 7] and ECS has the potential to be used for optical biopsy. An endoscope for ECS with a diameter equivalent to that of an ordinary endoscope is now commercially available and it has improved operability and is able to take more detailed images. Kumagai et al. [8] compared the latest ECS prototype with the older model and reported results showing its usefulness, especially for the diagnosis of ESCC. The new endoscope for ECS has not only a normal white-light imaging (WLI) mode but also a narrow band imaging (NBI) mode, making it possible to use it in daily clinical practice. However, there have only been retrospective studies on a comparison of ECS with histological diagnosis to determine whether precision endoscopic observation including ECS can be practically used for optical biopsy, and there has been no prospective study in which the diagnostic accuracy of ECS for lesions that have not yet undergone endoscopic biopsy and histological diagnosis were compared.

According to the 11th edition of the Japanese Classification of Esophageal Cancer [9], intraepithelial neoplasia (IN) is a low grade atypical neoplastic lesion corresponding to grade 3 histological diagnosis in the Vienna classification, and follow-up is recommended [6, 7, 10, 11]. Therefore, endoscopic diagnosis to differentiate ESCC that requires treatment from IN is very important. Since it has been reported that the ultra-high magnifying observation by ECS can focus from the superficial layer to a depth of 50 μm [12–14], we assume that differential diagnosis between IN and ESCC by ECS is possible. If precision endoscopic observation, including ECS, can substitute for biopsy tissue diagnosis, it will have various benefits in actual clinical practice.

We conducted a multicenter prospective study of surveillance for patients in a high-risk group of ESCC and evaluated the in vivo histological diagnostic accuracy of ECS for lesions that were suspected to be tumors.

## Methods

### Study design

This multicenter, exploratory, prospective study was conducted at Hokkaido University Hospital and Keiyukai Daini Hospital between September 1, 2019 and November 30, 2020. The ethics committee of each hospital approved the study protocol, and all participants gave written informed consent, based on the Helsinki Declaration (1964, 1975, amended in 1983, 2003, 2008 and 2013) of the World Medical Association. (UMIN Clinical Trials Registry ID: UMIN000037383).

### Patients

Patients who had been undergoing endoscopic follow-up at participating institutes after endoscopic resection for ESCC or treatment for head and neck squamous cell carcinoma (SCC) and who had multiple and various degrees of iodine-unstained areas in the esophagus on previous endoscopic images were regarded to be at high risk for ESCC and were eligible for this study. There patients were Grade C (more than 10 iodine-unstained areas per endoscopic view) in the JEC classification, which has been reported to be a high-risk group for metachronous multiple esophageal cancer [15]. Patients with lesions that were suspected to be early-stage ESCC who were referred to the participating institutions were also included in this study. Exclusion criteria were as follows: (1) patients who had already undergone biopsy histology and had a diagnosis of SCC, (2) patients who did not have a sufficient understanding after receiving an explanation for participation in this study, and (3) patients who the investigator considered were unsuitable as subjects.

### Procedures

Endoscopic surveillance by ECS was performed for patients who gave consent for participation in the study. The endoscopic examinations in this study were performed using a GIF-H290EC endoscope (Olympus Medical Systems Corp., Tokyo, Japan). This endoscope has a single integrated type lens reflection, 520-fold magnification with a focusing depth of 50 μm [12–14] and field of view of 570 × 500 μm [16, 17] and an outer diameter of 9.7 mm, channel diameter of 2.2 mm and length of 1030 mm.

WLI and NBI (with conventional magnification) were first performed. If there was a lesion that was suspected to be a tumor (e.g., redness in WLI and brownish area in NBI), the presence or absence of surface irregularity, background coloration or irregularity of the intrapapillary capillary loop, which are findings suggestive of malignancy, was determined. Then ultra-high magnifying observation was performed after staining the nucleus and cytoplasm using 2–4 ml of a mixture of 10 ml of 0.05% crystal violet and 1 ml of 1% methylene blue. We evaluated ECS based on the EC classification [18] described below (Additional file 1: Video 1). Lesions less than 5 mm in size were excluded from the study because it has been reported that these lesions are rarely cancerous [19, 20].

When the lesion was comprehensively diagnosed to be cancer by WLI, NBI and ultra-high magnifying observation, endoscopic resection was performed at a later date without biopsy. If the lesion was comprehensively diagnosed as non-cancerous, biopsy was performed. All endocytoscopic examinations were performed by 5 gastrointestinal endoscopists with expertise in endoscopic diagnosis of esophageal cancer (Shimoda Y, Shimizu Y, Takahashi H, Okahara S and Miyake T). Their periods of experience as gastrointestinal endoscopists were 5 years, 30 years, 24 years, 18 years and 4 years, respectively.

### EC classification

**Fig. 1:**
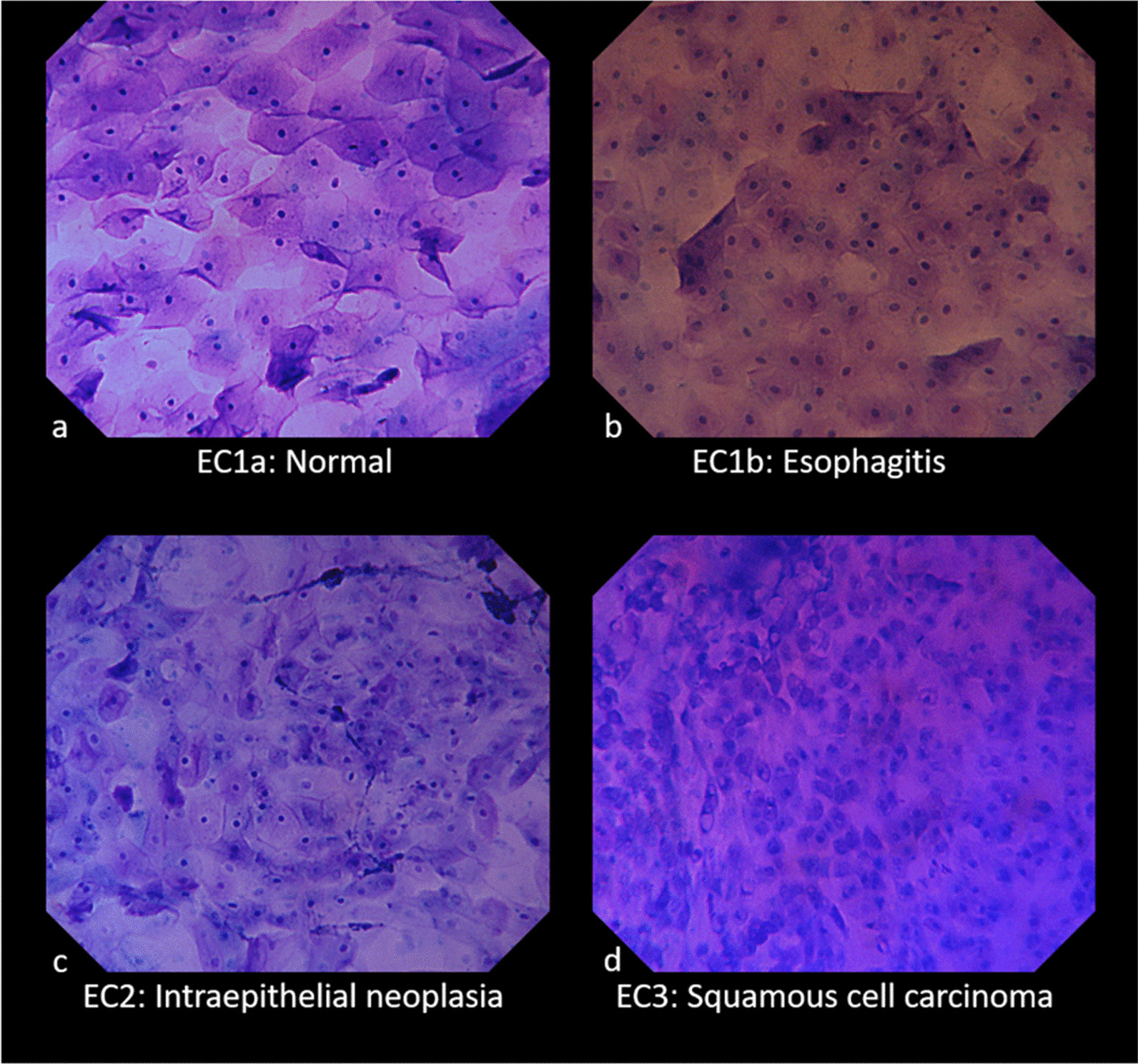
Esophageal EC classification. **a** EC1a (normal) shows regularly arranged large rhomboid-shaped cells. **b** EC1b (esophagitis) shows blunted edges and more rounded cells. **c** EC2 (intraepithelial neoplasia) shows an increase in cellular density but still with a recognizable cell structure. **d** EC3 (squamous cell carcinoma) shows complete loss of cellular structure with a significant increase in cellular density

In 2019, Inoue et al. reported an endocytoscopy classification (EC classification) [18] that is a modification of the aforementioned endocytoscopic atypia classification (ECA classification) [4] with a new generation of ECS. In this study, we used this classification. The details of the EC classification are shown below, and endocytoscopic images are presented in Fig. 1.

- EC1a is normal, showing regularly arranged large rhomboid-shaped cells.
- EC1b is esophagitis, showing blunted edges and more rounded cells.
- EC2 is intraepithelial neoplasia, showing an increase in cellular density but still with a recognizable cell structure.
- EC3 is squamous cell carcinoma, showing complete loss of cellular structure with a significant increase in cellular density.

### Histologic analysis

Endoscopic submucosal dissection (ESD) specimens were paraffin-embedded, cut into longitudinal slices of 2 mm in width, and stained with hematoxylin–eosin. Biopsy specimens were paraffin-embedded, cut in the centers, and stained with hematoxylin–eosin. The pathologist, who was blinded to the clinical characteristics of the patients, made the diagnosis based on the 11th edition of the Japanese Classification of Esophageal Cancer [9].

### Endpoints

The primary endpoint was the sensitivity of ECS diagnosis, defined as the proportion of lesions diagnosed as SCC histologically among those with evaluable images that were comprehensively diagnosed as carcinoma by endoscopic observation. The secondary endpoints were the percentage of evaluable endocytoscopic images, specificity of diagnosis by ECS, positive predictive value (PPV), negative predictive value (NPV), and accuracy.

### Statistical analysis

In a previous study, the sensitivity of endoscopic biopsy for early-stage ESCC was reported to be 85.6% [21]. Assuming an expected sensitivity of 95% for malignant diagnosis by ECS in this study and a threshold sensitivity of 80%, the number of esophageal cancer cases to yield 80% power at a significance level of 5% (one-sided) was calculated to be 35 cases based on the binomial test (normal approximation). In addition, because the annual incidence of ESCC in the high-risk group of metachronous multiple ESCC was reported to be about 20% [15], the target number of patients was set at 200, taking into account the occurrence of dropouts and ineligible cases. The sensitivity, specificity, PPV, NPV, and accuracy of the diagnosis in ECS were calculated, including 95% confidence intervals. Statistical analyses were performed by JMP Pro 15.0.

## Results

### Study population

**Fig. 2:**
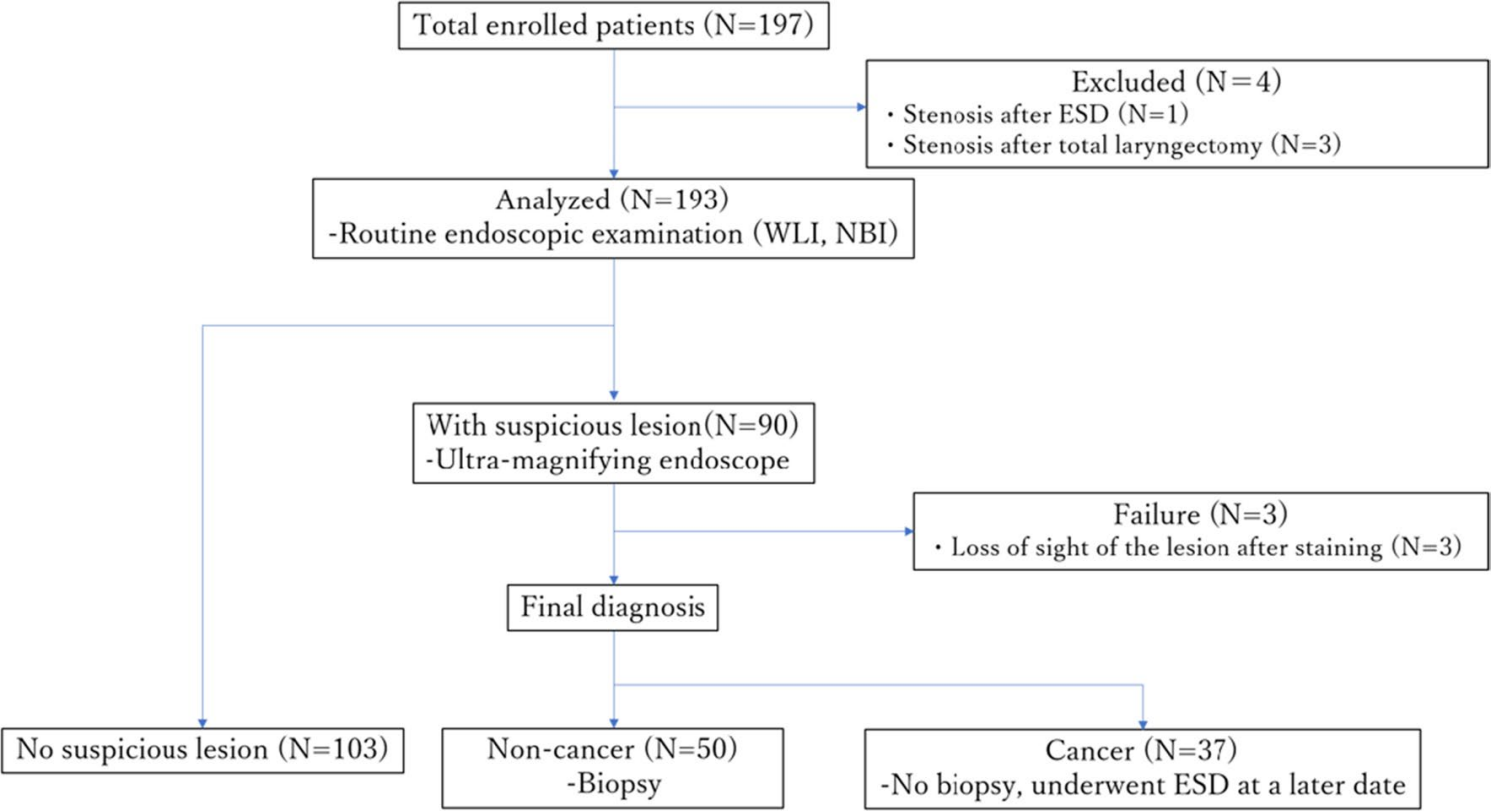
Flow chart showing diagnoses of the 197 lesions by using white-light imaging endoscopic, magnifying endoscopy combined with narrow-band imaging and ultra-high magnifying endoscopy. ESD, endoscopic submucosal dissection; *WLI* white-light imaging, *NBI* narrow-band imaging

A total of 197 patients were enrolled during the study period. Table 1 shows the characteristics of the patients. The patients included 167 men and 30 women with a median age of 73 years (range, 55–90 years). Regarding eligibility, there were 169 patients after endoscopic treatment for esophageal cancer, 80 patients after treatment for head and neck cancer (61 duplicated cases), and 9 patients referred for suspected esophageal cancer (without biopsy). Of the 197 patients enrolled, 4 were excluded because of inability to pass the endoscope due to stenosis after treatment (Fig. 2). After routine endoscopic examination, 90 patients were diagnosed with suspected tumorous lesions (including cancer and intraepithelial neoplasia) and underwent ultra-high magnifying observation. There were 3 failed cases in which ultra-high magnifying observation could not be performed because of loss of sight of the lesion after staining.

**Table 1:** Characteristics of the patients who were registered in the study

Sex, male/female	167/30
Age (years), Median (range)	73 (55–90)
*Background*	
After endoscopic resection for ESCC	169^†^
After treatment for HNSCC	80^†^
Referral cases (without biopsy)	9

*ESCC* esophageal squamous cell carcinoma, *HNSCC* head and neck squamous cell carcinoma

^†^Sixty one patients were duplicated

### Diagnostic outcome

The percentage of evaluable endocytoscopic images obtained in this study was 96.7% [95% confidence interval (CI), 90.8–98.9%].

**Fig. 3:**
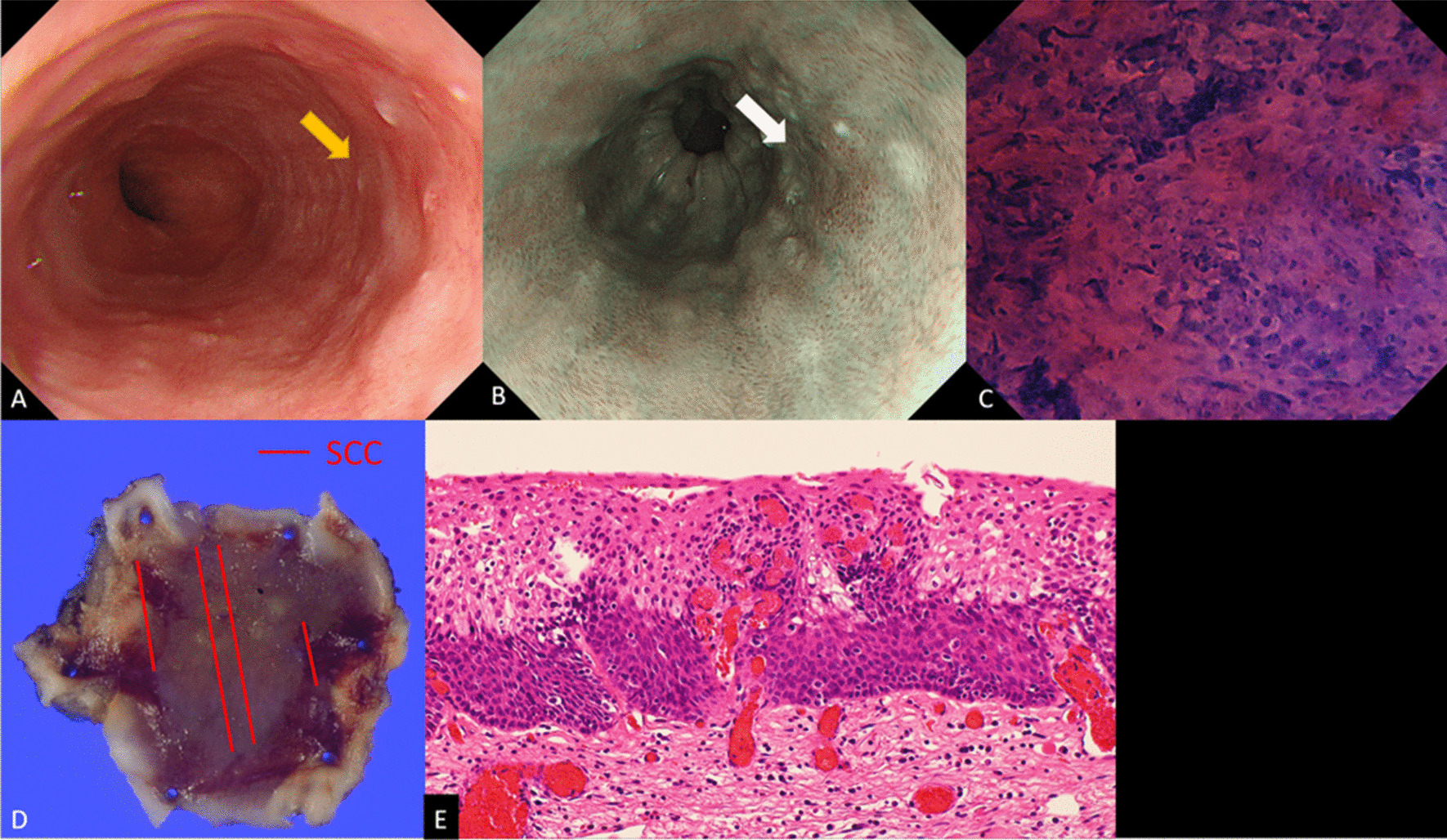
A representative case of SCC in EC3: **A** Endoscopic view by WLI. Redness and a slightly depressed lesion (yellow arrows) can be seen. **B** Endoscopic view by NBI. A brownish area with background coloration (white arrows) can be seen. **C** Endocytoscopic image of the lesion. EC classification was graded to be 3 by the endoscopist. **D** The resected specimen histologically revealed SCC (red line). **E** Photomicrograph of the resected specimen shows carcinoma in situ (H&E, orig. mag. × 40). SCC, squamous cell carcinoma; *EC3* EC classification 3, *WLI* white-light imaging, *NBI* barrow-band imaging

Table 2 shows the characteristics of 41 lesions in 37 patients who were comprehensively diagnosed with cancer and underwent ESD. The mean (± SD) tumor diameter was 15.4 ± 4.9 mm. One lesion was located in the cervical esophagus (Ce), 13 were located in the upper thoracic esophagus (Ut), 13 were located in the middle thoracic esophagus (Mt), 14 were located in the lower thoracic esophagus (Lt), and there was no lesion in the abdominal esophagus (Ae). In WLI, all of the lesions had redness and 17 lesions had surface irregularities. In NBI, all of the lesions had a brownish area and 38 lesions had background coloration. For the EC classification, there was no lesion classified as 1a, 1b, or 2, and all 41 lesions were classified as 3 **(**Fig. 3). ESD was performed in all patients at a later date, and the histopathological diagnosis of the resected specimens was SCC in all 41 lesions. The depths of tumor invasion were pT1a-EP in 14 lesions, pT1a-LPM in 25 lesions, and pT1a-MM in 2 lesions.

**Table 2:** Characteristics of the lesions that were diagnosed with cancer and underwent ESD

	41 Lesions (37 patients)
Lesions, size (mm), mean (± SD)	15.4 ± 4.9
Location, Ce/Ut/Mt/Lt/Ae	1/13/13/14/0
*Endoscopic findings (WLI)*	
Redness +/−	41/0
Surface irregular +/−	17/24
*Endoscopic findings (NBI)*	
Brownish area +/−	41/0
Background coloration +/−	38/3
*EC classification*	
1a/1b/2/3	0/0/0/41
*Histopathological diagnosis*	
Cancer/noncancer	41/0
*Depth of invasion*	
EP/LPM/MM	14/25/2

*ESD* endoscopic submucosal dissection, *Ce* cervical esophagus, *Ut* upper thoracic esophagus, *Mt* middle thoracic esophagus, *Lt* lower thoracic esophagus, *Ae* abdominal esophagus, *WLI* white light imaging, *NBI* narrow band imaging, *EP* epithelium, *LPM* lamina propria mucosae, *MM* muscularis mucosae

**Fig. 4:**
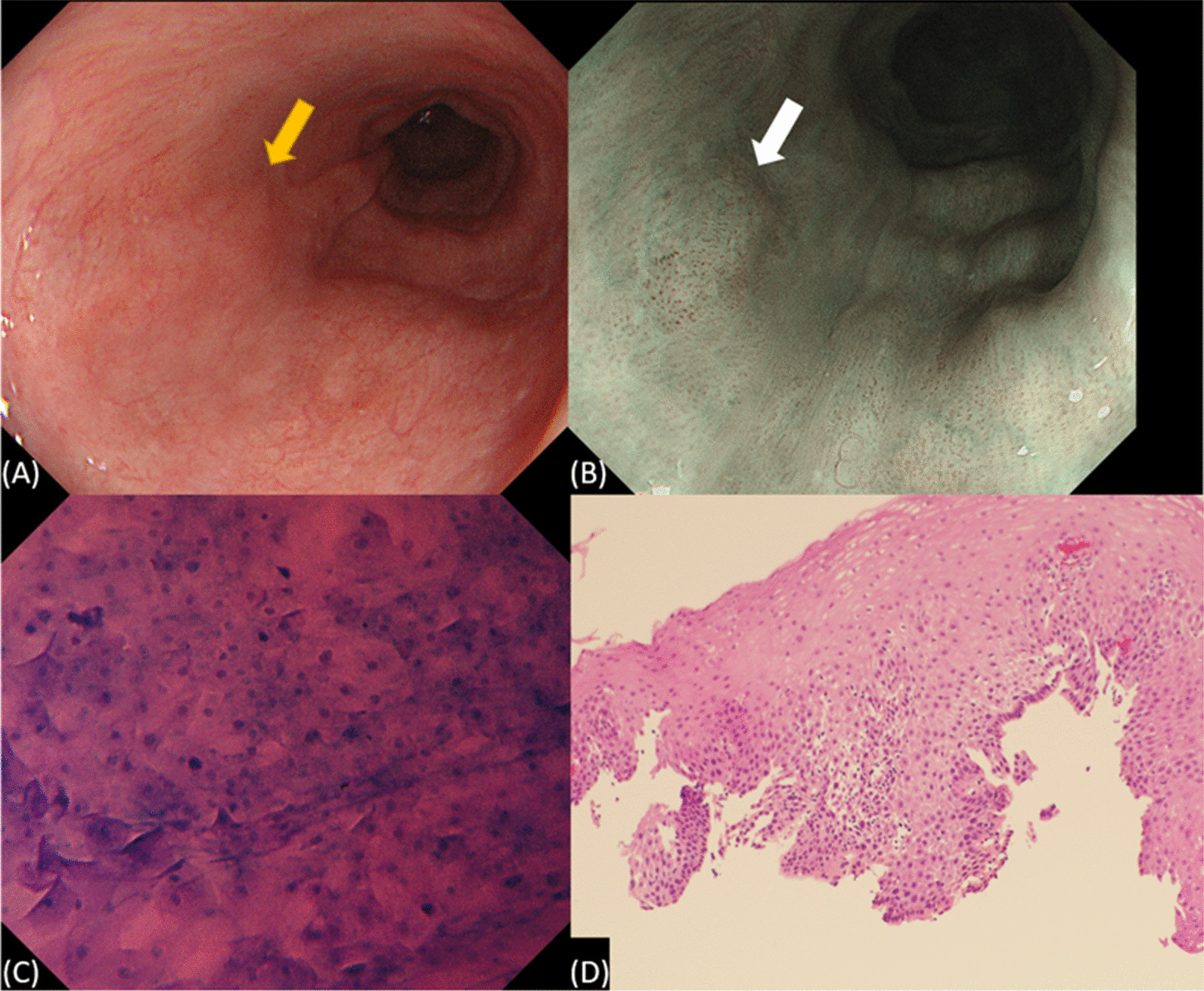
A representative case of intraepithelial neoplasia: **A** Endoscopic view by WLI. A faintly reddish lesion (yellow arrows can be seen. **B** Endoscopic view by NBI. A brownish area without background coloration (white arrows) can be seen. **C** Endocytoscopic image of the lesion. EC classification was graded to be 2 by the endoscopist. **D** Photomicrograph of the biopsied specimen shows atypical cells (H&E, orig. mag. × 40). *EC2* EC classification 2, *WLI* white-light imaging, *NBI* narrow-band imaging

Table 3 shows the characteristics of the 50 lesions in 50 patients comprehensively diagnosed as non-cancerous. The mean (± SD) lesion size was 9.6 ± 3.3 mm. Eighteen lesions were located in the Ut, 21 were located in the Mt, 10 were located in the Lt, and one lesion was located in the Ae. In WLI, 32 lesions had redness and 4 lesions had surface irregularities. In NBI, 47 lesions had a brownish area and 2 lesions had background coloration. For the EC classification, there was no lesion classified as 1a and 26 lesions were classified as 1b, 23 lesions were classified as 2 (Fig. 4), and one lesion was classified as 3. In that case of EC3, there were no WLI or NBI findings strongly suggestive of malignancy, but ultra-high magnifying showed EC3. It was a difficult case; however, the final comprehensive diagnosis of the lesion was non-cancer and a biopsy was performed. Histopathologically, only the EC3-rated lesion was diagnosed as SCC, while the other lesions were diagnosed as non-cancerous. Subclassification of noncancer was IN in 10 lesions, esophagitis in 31 lesions, and regenerative epithelium in 8 lesions.

**Table 3:** Characteristics of the lesions that were diagnosed as non-cancerous

	50 Lesions (50 patients)
Lesions, size (mm), mean	9.6 ± 3.3
Location, Ce/Ut/Mt/Lt/Ae	0/18/21/10/1
*Endoscopic findings (white light)*	
Redness +/−	32/18
Surface irregular +/−	4/46
*Endoscopic findings (NBI)*	
Brownish area +/−	47/3
Background coloration +/−	2/48
*EC classification*	
1a/1b/2/3	0/26/23/1
*Histopathological diagnosis*	
Cancer/noncancer	1/49
*Subclassification of noncancer*	
IN/Esophagitis/Regenerative ep	10/31/8

*Ce* cervical esophagus, *Ut* upper thoracic esophagus, *Mt* middle thoracic esophagus, *Lt* lower thoracic esophagus, *Ae* abdominal esophagus, *NBI* narrow band imaging, *IN* intraepithelial neoplasia, *ep* epithelium

Table 4 shows the diagnostic accuracy of ECS for each lesion. The sensitivity (95% CI) was 97.6% (87.7–99.7%), specificity (95% CI) was 100% (92.7–100%), diagnostic accuracy (95% CI) was 98.9% (94.0–99.8%), PPV (95% CI) was 100% (91.4–100%) and NPV (95% CI) was 98.0% (89.5–99.7%).

**Table 4:** Accuracy of endoscopic diagnosis for esophageal lesions by using ECS (Per lesion)

Sensitivity (%)^†^	Specificity (%)^†^	Accuracy (%)^†^	PPV (%)^†^	NPV (%)^†^
97.6(87.7–99.7)	100(92.7–100)	98.9(94.0–99.8)	100(91.4–100)	98.0(89.5–99.7)

*ECS* endocytoscopy, *PPV* positive predictive value, *NPV* negative predictive value

^†^Values are given as % (95% confidence interval)

## Discussion

Unlike early-stage colorectal cancer and colorectal adenoma, which are often observed as findings of elevated lesions, early-stage upper gastrointestinal cancers such as early-stage ESCC and early-stage gastric carcinoma are often observed as findings of flat or slightly depressed lesions, and the indication for treatment is therefore generally determined after direct endoscopic biopsy and definitive diagnosis as cancer. However, in early-stage ESCC, correct histological diagnosis may not be obtained because of the fact that IN is often mixed in the lesion [22] and the biopsy tissue may be damaged. The accuracy of biopsy diagnosis for early-stage esophageal cancer has been reported to be about 90%, the same as that of precise endoscopic diagnosis [2]. While performing many biopsies can increase the diagnostic accuracy, it would increase the risk of post-biopsy bleeding and fibrosis of the post-biopsy scar, which can make future endoscopic resections difficult. They are major clinical problems. Furthermore, in recent years, the number of patients taking antiplatelet and anticoagulant medications has been increasing, and the need for withdrawal of these medications is also an issue.

The results of our study showed an extremely high overall accuracy of 98.9%. It is noteworthy that there were no false positives in cases diagnosed and resected as SCC. One of the possible reasons for this good result is that ECS has the advantage of enabling observation and diagnose of all areas of the lesion by ultra-high magnification, whereas biopsy only enable diagnosis of a single point in the lesion. Ultra-high magnifying observation is considered to be highly reliable, and if optical biopsy can be put into practical use, it will not only solve the aforementioned problems but also contribute to a reduction in the burden on pathologists. As for the one false negative case that was comprehensively diagnosed to be IN but confirmed to be SCC by biopsy histopathology, that was a case in which the pathologist could not decide with high confidence whether it was IN or SCC. It is considered that cases in which diagnosis by pathology is difficult would be also difficult to diagnose by ECS.

The most important weaknesses of ECS diagnosis revealed in this study was that there were three cases in which the lesions became obvious after staining and no endocytoscopic images of them could be obtained. These were all small lesions, less than 1 cm in diameter, that were suspected to be non-cancerous by WLI or NBI, and they were found to be non-neoplastic by subsequent follow-up endoscopy. On the other hand, it was not difficult to identify suspected cancerous lesions diagnosed to be EC3 after staining, even if they were small lesions, because the entire lesion was stained darkly, reflecting the enlarged and dense nuclei after staining. We consider that the most efficient way to use ECS in clinical practice is to stain lesions suspected to be ESCC by WLI and NBI and perform an optical biopsy with ultra-high magnifying observation as the final confirmation.

There are some limitations in this study. First, the lesions were comprehensively diagnosed to be cancer or non-cancer by WLI, NBI and ultra-high magnifying observation, and we therefore cannot evaluate the exact additional effect of ultra-high magnifying observation alone. The procedure time for ultra-high magnifying observation takes a few minutes. However, performing ultra-high magnifying observation can provide cellular imaging (which cannot be obtained by NBI magnification) without biopsy and would reduce the task of pathologists. We consider that the difference in the required additional time for endoscopic biopsy and ultra-high magnifying observation would be small. Second, all of the investigators in this study were endoscopists with expertise in endoscopic diagnosis of early-stage esophageal cancer, and it is unclear whether comparable results are possible for general endoscopists. However, in recent years, artificial intelligence (AI) has been put to practical use in various fields of medicine, and its application is also being attempted in the field of gastrointestinal endoscopic diagnosis [23–27]. For lower gastrointestinal endoscopic diagnosis, a device that combines ECS with AI has been developed, and it has been shown by a multicenter retrospective study that the differential diagnosis ability between adenomas and non-adenomas is extremely high [28]. The device has become commercially available and multicenter prospective studies are underway. In addition, a retrospective study in which AI was used for diagnosis of endocytoscopic images by ECS has been conducted for early-stage ESCC, and the overall accuracy in that study was 90.9% [29]. If a comprehensive diagnostic system for WLI, NBI, and endocytoscopic images is developed in the future, it is expected that novice endoscopists will be able to obtain the same diagnostic ability as that in this study.

## Conclusions

In conclusion, based on the result of this prospective study, optical biopsy by using ECS for lesions that are suspected to be tumorous by WLI and NBI is considered to be sufficient in clinical practice.

## Supplementary Information

**Additional file 1: Video 1.** Endocytoscopy was performed on a lesion suspected to be cancerous by white light imaging and narrow band imaging, which was evaluated as EC3. Endoscopic submucosal dissection was performed later. Histopathologically, the lesion was diagnosed as squamous cell carcinoma.

## Data Availability

The datasets generated or analyzed during the current study are not publicly available due to limitations of ethical approval involving the patient data and anonymity but are available from the corresponding author on reasonable request.

## Abbreviations

ECS: Endocytoscopy
ESCC: Esophageal squamous cell carcinoma
WLI: White-light imaging
NBI: Narrow band imaging
IN: Intraepithelial neoplasia
SCC: Squamous cell carcinoma
EC classification: Endocytoscopy classification
ECA classification: Endocytoscopic atypia classification
ESD: Endoscopic submucosal dissection
PPV: Positive predictive value
NPV: Negative predictive value
SD: Standard deviation
Ce: Cervical esophagus
Ut: Upper thoracic esophagus
Mt: Middle thoracic esophagus
Lt: Lower thoracic esophagus
Ae: Abdominal esophagus
AI: Artificial intelligence
